# Total Neoadjuvant Therapy Versus Neoadjuvant Chemoradiation for Locally Advanced Rectal Cancer: A Multi-Institutional Real-World Study

**DOI:** 10.3390/cancers16183213

**Published:** 2024-09-21

**Authors:** Elif Şenocak Taşçı, Arda Ulaş Mutlu, Onur Saylık, Ömer Fatih Ölmez, Ahmet Bilici, Erdem Sünger, Osman Sütçüoğlu, Ömür Berna Çakmak Öksüzoğlu, Nuriye Özdemir, Orhun Akdoğan, İbrahim Vedat Bayoğlu, Nargiz Majidova, Ali Kaan Güren, Esra Özen Engin, İlhan Hacıbekiroğlu, Özlem Er, Faysal Dane, Mustafa Bozkurt, Esra Turan Canbaz, Sibel Erdamar, Erman Aytaç, Leyla Özer, İbrahim Yıldız

**Affiliations:** 1 Department of Medical Oncology, Kanuni Sultan Süleyman Training and Research Hospital, 34295 Istanbul, Turkey; 2 Department of Medicine, Acıbadem MAA University, 34560 Istanbul, Turkey; 3 Department of General Surgery, Acıbadem MAA University, 34560 Istanbul, Turkey; onur.saylik@acibadem.com (O.S.); erman.aytac@acibadem.com (E.A.); 4 Department of Medical Oncology, Medipol University Faculty of Medicine, 34815 Istanbul, Turkeyabilici@medipol.edu.tr (A.B.);; 5 Department of Medical Oncology, Etlik City Hospital, 06010 Ankara, Turkey; 6 Department of Medical Oncology, Gazi University Faculty of Medicine, 06560 Ankara, Turkey; nuriyeozdemir@gazi.edu.tr (N.Ö.); orhunakdogan@gazi.edu.tr (O.A.); 7 Department of Medical Oncology, Marmara University Faculty of Medicine, 34722 Istanbul, Turkey; 8 Department of Medical Oncology, Sakarya University Training and Research Hospital, 54187 Sakarya, Turkey; 9 Department of Medical Oncology, Acıbadem MAA University, 34560 Istanbul, Turkey; ozlem.er@acibadem.com (Ö.E.); leyla.ozer@acibadem.com (L.Ö.); ibrahim.yildiz@acibadem.com (İ.Y.); 10 Department of Medical Oncology, Acıbadem Altunizade Hospital, 34660 Istanbul, Turkey; faysal.dane@acibadem.com; 11 Department of Medical Oncology, Acıbadem Atakent Hospital, 34660 Istanbul, Turkey; mustafa.bozkurt@acibadem.com; 12 Department of Medical Oncology, Bakırköy Dr. Sadi Konuk Training and Research Hospital, 34147 Istanbul, Turkey; 13 Department of Pathology, Acıbadem MAA University, 34560 Istanbul, Turkey; sibel.erdamar@acibadem.com

**Keywords:** total neoadjuvant therapy, induction, consolidation, chemoradiotherapy, pathological complete response, locally advanced rectal cancer

## Abstract

**Simple Summary:**

Real-world studies comparing TNT and CRT are vital for advancing the treatment of LARC. Our study provides valuable insights reflecting the diverse patient populations and varied clinical practices encountered and demonstrates the advantage of TNT, providing a superior alternative to standard CRT and potentially enhancing treatment outcomes and quality of life.

**Abstract:**

Total neoadjuvant therapy (TNT) has emerged as a promising approach for managing locally advanced rectal cancer (LARC), aiming to enhance resectability, increase pathological complete response (pCR), improve treatment compliance, survival, and sphincter preservation. This study compares the clinical outcomes of TNT, with either induction or consolidation chemotherapy, to those of the standard chemoradiotherapy (CRT). In this retrospective multi-institutional study, patients with stage II-III LARC who underwent CRT or TNT from seven oncology centers between 2021 and 2024 were retrospectively analyzed. The TNT group was categorized into induction or consolidation groups based on the sequence of chemotherapy and radiotherapy. Clinical and pathological data and treatment outcomes, including pCR, event-free survival (EFS), and overall survival (OS), were analyzed. Among the 276 patients, 105 received CRT and 171 underwent TNT. The TNT group showed significantly higher pCR (21.8% vs. 2.9%, *p* < 0.001) and lower lymphatic (26.3% vs. 42.6%, *p* = 0.009), vascular (15.8% vs. 32.7%, *p* = 0.002), and perineural invasion rates (20.3% vs. 37.6%, *p* = 0.003). Furthermore, 16.9% of TNT patients opted for non-operative management (NOM), compared to 0.9% in the CRT group (*p* < 0.001). The median interval between the end of radiotherapy and surgery was longer in the TNT group (17.6 weeks vs. 8.8 weeks, *p* < 0.001). The 3-year EFS was 58.3% for CRT and 71.1% for TNT (*p* = 0.06). TNT is associated with higher pCR, lower lymphatic and vascular invasion rates, and higher rates of NOM compared to CRT. This supports the use of TNT as a viable treatment strategy for LARC, offering potential benefits in quality of life.

## 1. Introduction

Challenges in the management of locally advanced rectal cancer (LARC), which accounts for 5–10% of all rectal cancer cases, have prompted the adoption of a multimodal therapy approach [1]. Preoperative long-course chemoradiotherapy (LCRT) or short-course radiotherapy (SCRT), both followed by total mesorectal excision (TME) with or without adjuvant chemotherapy, have been the standard of care for stage II–III rectal cancer over the past decades [2]. While SCRT and LCRT have shown comparable survival and local control rates, LCRT has demonstrated superior local recurrence and pathological complete response (pCR) rates [2,3]. In specialized centers, local recurrence rates have improved to 5–8% [4]. Despite these advances, 30–40% of patients still develop distant metastases [5]. This prompted the use of adjuvant chemotherapy, though its effectiveness was limited. The reduced efficacy of adjuvant chemotherapy has been attributed to factors like delays, treatment compliance, and postoperative complications [6]. Despite these advancements, distant recurrence rates and long-term survival outcomes continued to necessitate new treatment strategies and resulted in the emergence of total neoadjuvant therapy (TNT).

TNT involves the administration of oxaliplatin-based systemic chemotherapy and radiotherapy prior to surgery, aiming to downsize the tumor and reduce the risks of local recurrence and the incidence of occult micrometastases. Recent studies have demonstrated the superiority of TNT over traditional neoadjuvant protocols in terms of pCR rates and improved long-term oncological outcomes [7,8]. In addition, the “watch-and-wait” approach, which focuses on organ preservation, has gained considerable attention as an alternative to immediate surgery following TNT in carefully selected patients [9]. 

Current TNT trials feature varied inclusion criteria, making it challenging to determine the optimal sequence for radiotherapy (SCRT or LCRT) and systemic chemotherapy [10]. Unlike earlier studies that evaluated SCRT and LCRT, limited studies have compared induction and consolidation chemotherapy within the context of TNT. One suggested more rectal preservation with consolidation chemotherapy by enhancing sustained clinical complete response rates [11]. Nevertheless, information on the ideal sequence to increase pCR rates and decrease distant metastases is still lacking. 

Real-world observational studies have increasingly demonstrated their value as a tool for evaluating the benefits and limitations of guideline-recommended therapies within more representative patient populations [12]. Such investigations offer essential insights into treatment outcomes in less controlled clinical settings [13]. This study aims to compare the clinical outcomes of TNT, incorporating either induction or consolidation chemotherapy, with those of the standard CRT approach in Turkish patients with stage II-III LARC. 

## 2. Materials and Methods

### 2.1. Study Design and Participants

In this multi-institutional retrospective study (IRB number: 2024–2/53), patients diagnosed with LARC from 2021 to 2024 in seven oncology centers were analyzed. The inclusion criteria were as follows: individuals aged 18 years who had undergone either standard neoadjuvant CRT before surgical resection or TNT because of LARC (cT3/T4 or T_any_N+). Patients who did not complete the planned treatment (due to adverse events or disease progression or own choice), received prior pelvic radiation, or had concurrent primary malignancy were excluded. The demographic and clinical characteristics of the patients, pathological features, details of neoadjuvant treatment, data on treatment outcomes such as pCR, completeness of total mesorectal excision (TME), and follow-up and survival data were obtained from medical records.

### 2.2. Treatment Modalities

All included patients underwent comprehensive staging through contrast-enhanced computed tomography (CT), magnetic resonance imaging (MRI) of the pelvis, and/or 18-fluorodeoxyglucose positron emission tomography (FDG-PET) scans prior to treatment to exclude distant metastases. Patients were categorized based on the type of neoadjuvant treatment they received before surgical resection. The standard CRT group all received fluoropyrimidine-based LCRT over a 5-week period, followed by surgery and a total of 4 months of adjuvant chemotherapy with either FOLFOX (folinic acid, fluorouracil, and oxaliplatin) or CAPOX (capecitabine and oxaliplatin). Nineteen patients in the CRT group received one or two additional cycles of chemotherapy during the waiting interval between completing radiotherapy and surgery. The TNT group received a combination of fluoropyrimidine-based LCRT or SCRT (5 × 5 Gy) followed by systemic chemotherapy or vice versa, according to the treatment protocol of the respective center. Systemic chemotherapy regimens, determined by institutional protocols, included either FOLFOX, FOLFIRINOX (folinic acid, fluorouracil, irinotecan and oxaliplatin), or CAPOX. The TNT group was further subdivided based on the treatment sequence. The induction group included patients who received systemic chemotherapy before radiotherapy, whereas the consolidation group received radiotherapy first. 

Clinical complete response (cCR) was defined as the absence of residual tumors following pelvic MRI or FDG-PET and no detectable viable tumor cells found from the biopsy via rectosigmoidoscopy. Following the completion of therapy, patients either underwent TME or were managed with the watch-and-wait approach. Pathological evaluation was performed by experienced pathologists. Tumor regression was assessed using the CAP (College of American Pathologists) tumor regression grading system [14]. The CAP system grades tumor response to therapy as follows: grade 0—no viable cancer cells (complete response); grade 1—single cells or rare small groups of cancer cells (near-complete response); grade 2—residual cancer with evident tumor regression but more than single cells or rare small groups of cancer cells (partial response); and grade 3—extensive residual cancer with no evident tumor regression (poor or no response). In the watch-and-wait approach, patients with cCR were closely monitored without immediate surgery. The follow-up was conducted at 12-week intervals for 2 years and at 24-week intervals for the following 3 years according to NCCN guidelines for all groups by all institutions [15].

### 2.3. Statistical Analyses

Data were analyzed using SPSS (Statistical Package for the Social Sciences) version 26.0 (IBM Corp., Armonk, NY, USA). Categorical variables were compared using the chi-square test or Fisher’s exact test, as appropriate. The primary objective was to compare pCR and pathological outcomes (lymphatic and vascular invasion rates) between the TNT and CRT groups, as well as within the induction and consolidation TNT subgroups. The secondary objective was to investigate the rates of non-operative management (NOM) and event-free survival (EFS) in both groups. EFS was calculated from the initiation of treatment until the occurrence of disease recurrence (local or metastatic) or death. OS was calculated from the initiation of treatment until the last follow-up or death. All statistical tests were two-sided, and a *p*-value of <0.05 was considered statistically significant. 

## 3. Results

### 3.1. Patient Characteristics 

A total of 276 patients were included in the study. Of these, 105 (38%) were in the CRT and 172 (62%) were in the TNT group. The median age was 60.1 ± 13.5 years for the CRT group and 56 ± 11.3 years for the TNT group. The two groups had comparable characteristics, except for the tumor grade (*p* = 0.025). Furthermore, 35.1% of the patients were females and 64.9% were male, with no significant difference between the groups. In terms of clinical staging, 73.2% of patients were classified as cT2–3, with 70.2% in the TNT group and 78.2% in the CRT group (*p* = 0.149). Regarding the clinical N stage, 85.9% were classified as cN1–2, with 87.7% in the TNT group and 82.9% in the CRT group (*p* = 0.26). Mismatch repair protein (MMR) status revealed that 96.3% of patients had proficient MMR, with 94.1% in the TNT group and 99.1% in the CRT group (*p* = 0.07). A significant difference was noted in tumor differentiation, with 85.4% of tumors being well-to-moderately differentiated overall, with a higher proportion in the CRT group (91.1%) than in the TNT group (73.6%) (*p* = 0.025). The majority of patients received capecitabine concurrently with radiotherapy (RT). Regarding the RT course, 86.6% underwent LCRT, with a slightly higher proportion in the CRT group (89.4%) than in the TNT group (84.8%) (*p* = 0.275). 

In the TNT group, the number of chemotherapy cycles was six (range 3–12). In the CRT group, during the waiting period until surgery, only nineteen patients received a median of two cycles (range 1–5) of chemotherapy. The median interval between the end of RT and surgery was significantly longer in the TNT group (17.6 weeks) than in the CRT group (8.8 weeks) (*p* < 0.001). Post-neoadjuvant treatment (NAT) approaches showed that 16.9% of TNT patients opted for NOM compared with 0.9% in the CRT group (*p* < 0.001). Two patients from the NOM group eventually underwent surgery, and one patient developed lung metastases. The detailed demographic and clinicopathological characteristics of the patients are presented in Table 1.

In the TNT group, 102 (59.6%) patients were in the consolidation group and 69 (40.3%) were in the induction group. The two groups had similar demographic and pathologic characteristics, except for the tumor grade (*p* = 0.03). The majority of patients in both groups received LCRT, with capecitabine use being significantly more common in the consolidation group. The detailed patient characteristics and treatment outcomes of the TNT group are shown in Table 2.

### 3.2. Treatment Outcomes

Most of the patients underwent surgery, with 83.1% in the TNT group and 99.1% in the CRT group. Open surgery was performed in 28.2% of the patients, whereas 71.8% underwent minimally invasive procedures. Laparoscopic surgery was more common in the TNT group (80.4%) than in the CRT group (64.9%), whereas robotic surgery was more prevalent in the CRT group (35.1%) than in the TNT group (18.7%). TME status was similar across groups, with 91.5% of patients achieving TME.

pCR was significantly higher in the TNT group (21.8%) than in the CRT group (2.9%) (*p* < 0.001). ypT3 stage was more common in CRT patients (65.1%) than in TNT patients (44.4%). The pathological N category showed ypN0 in 73.9% of TNT patients and 64.1% of CRT patients (*p* = 0.097). Lymphatic, vascular, and perineural invasion were significantly higher in the CRT group compared to TNT group (42.6% vs. 26.3% (*p* = 0.009), 32.7% vs. 15.8% (*p* = 0.002), and 37.6% vs. 20.3% (*p* = 0.003), respectively). The median number of harvested lymph nodes was significantly higher in the CRT group than in the TNT group (*p* < 0.001). The complete resection (R0) rates were also similar in the two groups (97.6% vs. 95.2%). In the TNT group, four patients had positive surgical margins, while in the CRT group, five had positive surgical margins. The treatment outcomes for both groups are summarized in Table 3.

The median duration of follow-up was 72 months for both groups. The recurrence rate was 17.5% (30/171 patients) in the TNT group, while it was 27.6% (29/105 patients) in the CRT group. Most of the recurrences were distant. Local recurrence was detected in five and six patients in the TNT and CRT groups, respectively. The 3-year EFS was 58.3% for CRT and 71.1% for TNT groups (*p* = 0.06). There were eight events associated with OS, with five in the TNT and three in the CRT groups. The number of deaths was not sufficient to analyze survival. There were eight events among NOM patients: two in CRT, three in induction, and three in consolidation groups. Regrowth was observed in six patients who were followed with NOM, while lung metastases were observed in two patients without regrowth during follow-up. TNT was associated with higher rates of NOM, better pathological response, and lower rates of lymphatic and vascular invasion compared to CRT, whereas survival outcomes were similar between the two treatment modalities.

Regarding TNT group, post-NAT approach showed that most patients underwent surgery, with no significant difference in the type of surgical approach. Pathologic T and N categories, lymphatic invasion, and vascular invasion rates were comparable between the groups. The tumor regression grading (TRG) and pCR rates were similar, with no significant differences observed. The median number of chemotherapy cycles was six in both groups. Overall, both consolidation and induction cohorts demonstrated similar clinical and pathological outcomes, with minor variations in specific areas (Table 2).

Grade 3–4 treatment-related adverse events occurred in 38% of patients in the TNT group and 26% in the CRT group. The most common grade 3–4 adverse events in the TNT group were hematologic toxicities (16%), diarrhea (15%), and neuropathy (5%), while in the CRT group, they were gastrointestinal side effects (15%) and dermatitis (10%). There were no treatment-related toxic deaths in either group. Additionally, there was no significant difference in treatment-related adverse events between the induction and consolidation arms within the TNT group.

EFS did not differ significantly between the two groups. The median EFS was 22.7 months (range 16.4–26.5) for the consolidation group and 18.8 months (range 16.5–24.8) for the induction group (*p* = 0.507). There were 18 events out of 102 in consolidation and 12 out of 69 events in induction. Additionally, two patients in consolidation and two in induction died during the analysis.

## 4. Discussion

In this study, we evaluated the effectiveness of TNT versus standard CRT in treating LARC, particularly focusing on pathological outcomes and survival rates. Our retrospective multi-institutional analysis provides strong evidence supporting the use of TNT, showing that it improves pCR rates, reduces lymphatic and vascular invasion, and promotes NOM compared with CRT. 

The TNT group showed better pCR rates, consistent with prior studies and meta-analyses [7,8,16,17]. The improved pCR rates are crucial as they are associated with better long-term outcomes, including lower recurrence rates and improved survival. Key trials such as STELLAR (four cycles of CAPOX with SCRT), RAPIDO (SCRT and six cycles of CAPOX), and UNICANCER-PRODIGE 23 (FOLFIRINOX and LCRT) have demonstrated these benefits [7,8,18]. Recently, Wang et al. compared a new standardized TNT approach (LCRT with six cycles of neoadjuvant CAPOX (one cycle of induction CAPOX, two cycles of concurrent CAPOX, and three cycles of consolidation CAPOX)) with neoadjuvant CRT and showed improved pCR and EFS compared to the standard approach [19].

The median interval between the end of RT and surgery was significantly longer in the TNT group (17.6 weeks) than in the CRT group (8.8 weeks). Extending the interval between the completion of CRT and surgery can potentially increase the likelihood of achieving pCR. The optimal time frame often ranges between 6 and 12 weeks, with some evidence suggesting that longer intervals allow for enhanced tumor regression and better surgical outcomes [11]. However, prolonged waiting periods must be carefully balanced against the risk of tumor progression or complications. This phenomenon is also established in the TNT group. Some studies revealed that upfront LCRT followed by consolidation chemotherapy resulted in higher pCR and organ preservation rates [10,13]. The CAO/ARO/AIO-12 trial [13] reported similar 3-year DFS, LRR, and distant metastasis rates between induction and consolidation chemotherapy, but higher pCR rates in the consolidation group (25% vs. 17%, *p* < 0.001). The trial showed similar DFS and OS regardless of the sequence, but higher TME-free survival with the CRT-first approach (54% vs. 39% at 5 years) [20]. Therefore, preferring consolidation chemotherapy may be a more appropriate strategy to achieve pCR after TNT for the watch-and-wait approach or local excision planned patients. However, in our study, there was no difference in pCR and EFS between the induction and consolidation groups.

Real-world studies have also evaluated TNT versus CRT. Yu et al.’s large retrospective study found similar pCR rates between TNT and CRT but no overall survival advantage, likely due to heterogeneous data and limited TNT protocol details [21]. Conversely, Turfa et al. found doubled pCR rates and a 3-year EFS and OS advantage with TNT in a real-world setting [22]. Our outcomes align with those of controlled trials but must be interpreted cautiously due to potential differences in patient ethnicity, study design, and other confounding factors. In addition to pCR, the improved pathological outcomes in the TNT group, i.e., lower ypT3 prevalence and reduced lymphatic, vascular, and perineural invasion, support the efficacy of TNT. 

According to our study, TNT does not affect the type of surgery. While the rate of laparoscopic surgery was observed to be higher in the TNT group, the rates of open surgery and minimally invasive surgery were similar across both groups. In contrast to our findings, the OPRA study reported a lower rate of R1 resection in the TNT group compared to the CRT group [23]. Furthermore, the role of TNT in supporting organ preservation is emphasized by the increasing adoption of the “watch-and-wait” strategy for patients who attain cCR. The OPRA trial reported that 77% of patients with cCR were able to preserve their rectum over 3 years [11]. This is particularly important given the potential long-term functional and quality of life impacts of rectal surgery [24]. We found a significantly higher rate of NOM in the TNT group (16.9% vs. 0.9%, *p* < 0.001), likely due to higher pCR rates and better pathological responses, allowing patients to avoid surgery and its associated morbidities [25]. Therefore, TNT not only preserves sphincter function but also potentially improves the quality of life for patients who can avoid the morbidity associated with surgery [26]. Long-term data on the safety and efficacy of the watch-and-wait strategy are still evolving. However, the increased NOM percentages observed in the TNT cohort prove that TNT provides an advantage for the “watch-and-wait” strategy.

Though our study demonstrated improved pathological outcomes with TNT, the absence of significant survival differences raises questions about the long-term clinical impact of these improvements. These discrepancies may stem from variations in study design, patient populations, and treatment protocols. Additionally, the relatively short follow-up period and the small number of events could have affected survival outcomes. Although we did not find a significant difference in EFS between the consolidation and induction groups, the trend toward improved 3-year EFS in the TNT group suggests potential long-term benefits, consistent with findings from other recent studies [19].

Recent trials exploring immune checkpoint inhibitors in rectal cancer treatment have shown promising results. Neoadjuvant Dostarlimab proved its efficacy by achieving a 100% sustained cCR in patients with deficient MMR (dMMR) [27]. Avelumab was also implemented in the neoadjuvant setting, yielding promising response rates [28]. Long-term outcomes are still pending. Approximately 3% of rectal tumors are dMMR [29]. Our study also comprised 3.7% of patients with dMMR who were unable to receive immunotherapy in the neoadjuvant setting. These findings, combined with the evolving immunotherapy data, might increase cCR rates in the future.

One of the limitations of our study is its retrospective design which may have introduced selection bias. Additionally, variability in institutional protocols for chemotherapy and radiotherapy could affect patient outcomes. The follow-up period may also be insufficient to capture long-term survival benefits or late recurrence. Furthermore, excluding patients with incomplete treatment data may limit the generalizability of these findings.

## 5. Conclusions

Our study adds to the growing body of evidence supporting the use of TNT in rectal cancer treatment. The improved pathological responses and higher rates of NOM offer promising avenues for organ preservation and potentially less morbid treatment strategies. However, the lack of survival benefits in our study underscores the need for longer follow-up studies and larger prospective trials to fully elucidate the long-term benefits of TNT over CRT for rectal cancer treatment.

## Figures and Tables

**Table 1:** Baseline demographic and clinicopathological characteristics of the patients.

	Total	TNT	CRT	*p*
Age at Diagnosis (Years)	57.6 ± 12.3	56.1 ± 11.3	60.1 ± 13.5	**0.008**
Gender				
Male	179 (64.9)	104 (60.8)	75 (71.4)	0.073
Female	97 (35.1)	67 (39.2)	30 (28.6)	
Clinical T Stage				
T2–3	202 (73.2)	120 (70.2)	82 (78.2)	0.149
T4	74 (26.8)	51 (29.8)	23 (21.8)	
Clinical N Stage				
N0	39 (14.1)	21 (12.3)	18 (17.1)	0.26
N1–2	237 (85.9)	150 (87.7)	87 (82.9)	
Mismatch Repair Protein Status				
pMMR	211 (96.3)	110 (94.1)	101 (99.1)	0.07
dMMR	8 (3.7)	7 (5.9)	1 (0.9)	
Tumor differentiation				
Well differentiated (G1)/Moderately differentiated (G2)	181 (85.4)	89 (73.6)	92 (91.1)	***0.025***
Poorly differentiated (G3)	31 (14.6)	22 (26.4)	9 (8.9)	
Distance from Anal Verge				
<5 cm	86 (32.6)	52 (31.9)	34 (33.7)	0.66
5–10 cm	116 (43.9)	75 (46)	41 (40.6)	
≥10 cm	62 (23.5)	36 (22.1)	26 (25.7)	
Radiotherapy				
Short-course	37 (13.4)	26 (15.2)	11 (10.6)	0.275
Long-course	239 (86.6)	146 (84.8)	93 (89.4)	
Chemotherapy agent during CRT				
Capecitabine	205 (85.8)	121 (82.9)	84 (90.3)	0.108
5-FU infusion	34 (14.2)	25 (17.1)	9 (9.7)	
Chemotherapy agent				
CAPOX/FOLFOX	175 (92.6)	159 (93.5)	16 (84.2)	0.153
Other	14 (7.4)	11 (6.5)	3 (15.8)	
Time between RT and Surgery (Weeks)	12.9 (1.6–29.7)	17.6 (8.4–29.7)	8.8 (1.6–29.1)	***<0.001***

Abbreviations: TNT: total neoadjuvant treatment, CRT: chemoradiotherapy, pMMR: proficient mismatch repair, dMMR: deficient mismatch repair, FOLFOX: folinic acid, fluorouracil, and oxaliplatin, CAPOX: capecitabine and oxaliplatin.

**Table 2:** Characteristics and treatment outcomes of the TNT group.

	Total	Consolidation	Induction	*p*
Age at Diagnosis (Years)	56.1 ± 11.3	55.8 ± 11.4	56.4 ± 11.2	0.76
Gender				
Male	104 (60.8)	64 (62.7)	40 (57.9)	0.53
Female	67 (39.2)	38 (37.3)	29 (42.1)	
Clinical T Stage				
T2–3	120 (70.2)	76 (74.5)	44 (63.8)	0.132
T4	51 (29.8)	26 (25.5)	25 (36.2)	
Clinical N Stage				
N0	21 (12.3)	14 (13.7)	7 (10.1)	0.484
N1–2	150 (87.7)	88 (86.3)	62 (89.9)	
Mismatch Repair Status				
pMMR	110 (94.1)	65 (92.9)	45 (95.7)	0.519
dMMR	7 (5.9)	5 (7.1)	2 (4.3)	0.701
Tumor differentiation				
Well differentiated (G1)/Moderately differentiated (G2)	89 (80.2)	50 (73.5)	39 (88.6)	***0.03***
Poorly differentiated (G3)	22 (19.8)	18 (26.5)	4 (11.4)	
Distance from anal verge				
<5 cm	52 (31.9)	32 (32)	20 (31.7)	0.471
5–10 cm	75 (46)	43 (43)	32 (50.8)	
≥10 cm	36 (22.1)	25 (25)	11 (17.5)	
Radiotherapy				
Short-course	26 (15.2)	16 (15.7)	10 (14.3)	0.831
Long-course	146 (84.8)	86 (84.3)	60 (85.7)	
CRT Chemotherapy				
Capecitabine	121 (82.9)	78 (90.7)	43 (71.7)	***0.003***
5-FU infusion	25 (17.1)	8 (9.3)	17 (28.3)	
Chemotherapy Agent				
CAPOX/FOLFOX	159 (93.5)	94 (93.1)	65 (94.2)	>0.999
Other	11 (6.5)	7 (6.9)	4 (5.8)	
Chemotherapy Cycles	6 (3–12)	6 (3–12)	6 (4–12)	0.646
Surgery				
Yes	142 (83.1)	86 (84.3)	56 (81.2)	0.59
No	29 (16.9)	16 (15.7)	13 (18.8)	
Pathologic T category				
ypT0	31 (21.8)	18 (20.9)	13 (23.2)	0.426
ypT1	9 (6.3)	5 (5.8)	4 (7.2)	
ypT2	28 (19.7)	17 (10.8)	11 (19.6)	
ypT3	63 (44.4)	42 (48.8)	21 (37.5)	
ypT4	11 (7.8)	4 (4.7)	7 (12.5)	
Pathologic N category				
ypN0	105 (73.9)	63 (73.3)	42 (75)	0.817
ypN1–2	37 (26.1)	23 (26.7)	14 (25)	
Lymphatic invasion				
Yes	35 (26.3)	21 (26.3)	14 (26.4)	0.983
No	98 (73.7)	59 (73.7)	39 (73.6)	
Vascular invasion				
Yes	21 (15.8)	10 (12.5)	11 (20.8)	0.201
No	112 (84.2)	70 (87.5)	42 (79.2)	
Perineural invasion				
Yes	27 (20.3)	17 (21.3)	10 (18.9)	0.738
No	106 (79.7)	63 (78.7)	43 (81.1)	
Harvested LN (Median, Range)	16 (1–41)	16 (1–37)	16 (4–41)	0.927
TME Status				
TME	101 (90.9)	68 (90.7)	33 (9.7)	>0.999
Non-TME	10 (9.1)	7 (9.3)	3 (8.3)	
Tumor regression grading				
0	31 (22.9)	18 (21.7)	13 (23.5)	0.122
1	37 (27.4)	22 (26.5)	15 (29.4)	
2	60 (44.4)	39 (46.9)	21 (41.2)	
3	7 (5.3)	4 (4.9)	3 (5.9)	
pCR				
Yes	31 (21.8)	18 (20.9)	13 (23.2)	0.747
No	111 (78.2)	68 (79.1)	43 (76.8)	

Abbreviations: pMMR: proficient mismatch repair, dMMR: deficient mismatch repair, FOLFOX: folinic acid, fluorouracil, and oxaliplatin, CAPOX: capecitabine and oxaliplatin, LN: lymph node, TME: total mesorectal excision, pCR: pathologic complete response.

**Table 3:** Treatment outcomes in all cohorts.

	Total	TNT	CRT	*p*
**Surgery**				
Yes	245 (89.1)	142 (83.1)	103 (99.1)	***<0.001***
No	30 (10.9)	29 (16.9)	1 (0.9)	
**Grading of operative Specimen (TME)**				
Mesorectal plane (good)	134 (66.3)	77 (69.3)	57 (62.6)	0.422
Intramesorectal plane (moderate)	51 (25.2)	24 (21.6)	27 (29.7)	
Muscularis propria plane (poor)	17 (8.5)	10 (9.1)	7 (7.7)	
**Operation Approach**				
Open	69 (28.2)	40 (28.2)	29 (28.2)	0.998
Minimally invasive	176 (71.8)	102 (71.8)	74 (71.8)	
Laparoscopic	130 (73.9)	82 (80.4)	48 (64.9)	
Robotic	46 (25.5)	19 (18.7)	26 (35.1)	
**Pathologic T stage**				
ypT0	34 (13.9)	31 (21.8)	3 (2.9)	***<0.001***
ypT1	14 (5.7)	9 (6.3)	5 (4.9)	
ypT2	49 (20)	28 (19.7)	21 (20.4)	
ypT3	130 (53.1)	63 (44.4)	67 (65.1)	
ypT4	18 (7.3)	11 (7.8)	7 (6.7)	
**Pathologic N stage**				
ypN0	171 (69.8)	105 (73.9)	66 (64.1)	0.097
ypN1–2	74 (30.2)	37 (26.1)	37 (35.9)	
**Lymphatic invasion**				
Yes	78 (33.3)	35 (26.3)	43 (42.6)	***0.009***
No	146 (66.7)	98 (73.7)	58 (57.4)	
**Vascular invasion**				
Yes	54 (23.1)	21 (15.8)	33 (32.7)	***0.002***
No	180 (76.9)	112 (84.2)	68 (67.3)	
**Perineural invasion**				
Yes	65 (27.8)	27 (20.3)	38 (37.6)	***0.003***
No	169 (72.2)	106 (79.7)	63 (62.4)	
**Harvested LN (Median, Range)**	18.5 (1–63)	16 (1–41)	24 (5–63)	***<0.001***
**TME Status**				
TME	185 (91.5)	101 (90.9)	84 (92.3)	0.737
Non-TME	17 (8.5)	10 (9.1)	7 (7.7)	
**Tumor regression grade**				
0	34 (14.7)	31 (22.9)	3 (3.1)	***<0.001***
1	76 (32.9)	37 (27.4)	39 (40.6)	
2	114 (49.4)	60 (44.4)	54 (56.3)	
3	7 (3)	7 (5.3)	0 (0)	
**pCR**				
Yes	34 (13.9)	31 (21.8)	3 (2.9)	***<0.001***
No	211 (86.1)	111 (78.2)	100 (97.1)	

Abbreviations: TNT: total neoadjuvant treatment, CRT: chemoradiotherapy, LN: lymph node, TME: total mesorectal excision, CAP: pCR: pathologic complete response.

## Data Availability

The data that support the findings of this study are available on request from the corresponding author E.Ş.T.

## References

[1] Sung H., Ferlay J., Siegel R.L., Laversanne M., Soerjomataram I., Jemal A., Bray F. (2021). Global Cancer Statistics 2020: GLOBOCAN Estimates of Incidence and Mortality Worldwide for 36 Cancers in 185 Countries. CA Cancer J. Clin..

[2] Kapiteijn E., Marijnen C.A., Nagtegaal I.D., Putter H., Steup W.H., Wiggers T., Rutten H.J., Pahlman L., Glimelius B., van Krieken J.H. (2001). Preoperative radiotherapy combined with total mesorectal excision for resectable rectal cancer. N. Engl. J. Med..

[3] Folkesson J., Birgisson H., Pahlman L., Cedermark B., Glimelius B., Gunnarsson U. (2005). Swedish Rectal Cancer Trial: Long lasting benefits from radiotherapy on survival and local recurrence rate. J. Clin. Oncol..

[4] Påhlman L., Bohe M., Cedermark B., Dahlberg M., Lindmark G., Sjödahl R., Öjerskog B., Damber L., Johansson R. (2007). The Swedish rectal cancer registry. Br. J. Surg..

[5] Sauer R., Liersch T., Merkel S., Fietkau R., Hohenberger W., Hess C., Becker H., Raab H.-R., Villanueva M.-T., Witzigmann H. (2012). Preoperative Versus Postoperative Chemoradiotherapy for Locally Advanced Rectal Cancer: Results of the German CAO/ARO/AIO-94 Randomized Phase III Trial After a Median Follow-Up of 11 Years. J. Clin. Oncol..

[6] Sainato A., Nunzia V.C.L., Valentini V., Paoli A.D., Maurizi E.R., Lupattelli M., Aristei C., Vidali C., Conti M., Galardi A. (2014). No benefit of adjuvant Fluorouracil Leucovorin chemotherapy after neoadjuvant chemoradiotherapy in locally advanced cancer of the rectum (LARC): Long term results of a randomized trial (I-CNR-RT). Radiother. Oncol..

[7] Bahadoer R.R., Dijkstra E.A., van Etten B., Marijnen C.A.M., Putter H., Kranenbarg E.M., Roodvoets A.G.H., Nagtegaal I.D., Beets-Tan R.G.H., Blomqvist L.K. (2021). Short-course radiotherapy followed by chemotherapy before total mesorectal excision (TME) versus preoperative chemoradiotherapy, TME, and optional adjuvant chemotherapy in locally advanced rectal cancer (RAPIDO): A randomised, open-label, phase 3 trial. Lancet Oncol..

[8] Conroy T., Bosset J.F., Etienne P.L., Rio E., François É., Mesgouez-Nebout N., Vendrely V., Artignan X., Bouché O., Gargot D. (2021). Neoadjuvant chemotherapy with FOLFIRINOX and preoperative chemoradiotherapy for patients with locally advanced rectal cancer (UNICANCER-PRODIGE 23): A multicentre, randomised, open-label, phase 3 trial. Lancet Oncol..

[9] López-Campos F., Martín-Martín M., Fornell-Pérez R., García-Pérez J.C., Die-Trill J., Fuentes-Mateos R., López-Durán S., Domínguez-Rullán J., Ferreiro R., Riquelme-Oliveira A. (2020). Watch and wait approach in rectal cancer: Current controversies and future directions. World J. Gastroenterol..

[10] Ozer L., Yildiz I., Bayoglu V., Bozkurt M., Esen E., Remzi F.H., Gogenur I., Aytac E. (2021). Tailored total neoadjuvant therapy for locally advanced rectal cancer: One size may not fit for all!. Color. Dis..

[11] Verheij F.S., Omer D.M., Williams H., Lin S.T., Qin L.X., Buckley J.T., Thompson H.M., Yuval J.B., Kim J.K., Dunne R.F. (2024). Long-Term Results of Organ Preservation in Patients With Rectal Adenocarcinoma Treated With Total Neoadjuvant Therapy: The Randomized Phase II OPRA Trial. J. Clin. Oncol..

[12] Tang M., Pearson S.A., Simes R.J., Chua B.H. (2023). Harnessing Real-World Evidence to Advance Cancer Research. Curr. Oncol..

[13] Saesen R., Van Hemelrijck M., Bogaerts J., Booth C.M., Cornelissen J.J., Dekker A., Eisenhauer E.A., Freitas A., Gronchi A., Hernán M.A. (2023). Defining the role of real-world data in cancer clinical research: The position of the European Organisation for Research and Treatment of Cancer. Eur. J. Cancer.

[14] Stewart C.J., Hillery S., Plattell C. (2009). Protocol for the examination of specimens from patients with primary carcinomas of the colon and rectum. Arch. Pathol. Lab. Med..

[15] Benson A.B., Venook A.P., Al-Hawary M.M., Azad N., Chen Y.J., Ciombor K.K., Cohen S., Cooper H.S., Deming D., Garrido-Laguna I. (2022). Rectal Cancer, Version 2.2022, NCCN Clinical Practice Guidelines in Oncology. J. Natl. Compr. Canc Netw..

[16] Petrelli F., Trevisan F., Cabiddu M., Sgroi G., Bruschieri L., Rausa E., Ghidini M., Turati L. (2020). Total Neoadjuvant Therapy in Rectal Cancer: A Systematic Review and Meta-analysis of Treatment Outcomes. Ann. Surg..

[17] Zhang X., Ma S., Guo Y., Luo Y., Li L. (2022). Total neoadjuvant therapy versus standard therapy in locally advanced rectal cancer: A systematic review and meta-analysis of 15 trials. PLoS ONE.

[18] Jin J., Tang Y., Hu C., Jiang L.M., Jiang J., Li N., Liu W.Y., Chen S.L., Li S., Lu N.N. (2022). Multicenter, Randomized, Phase III Trial of Short-Term Radiotherapy Plus Chemotherapy Versus Long-Term Chemoradiotherapy in Locally Advanced Rectal Cancer (STELLAR). J. Clin. Oncol..

[19] Wang X., Liu P., Xiao Y., Meng W., Tang Y., Zhou J., Ding P.-R., Ding K.-F., Wang B., Guo Q. (2024). Total neoadjuvant treatment with long-course radiotherapy versus concurrent chemoradiotherapy in local advanced rectal cancer with high risk factors (TNTCRT): A multicenter, randomized, open-label, phase 3 trial. J. Clin. Oncol..

[20] Verheij F., Omer D., Williams H., Buckley J., Lin S., Qin L.X., Thompson H., Yuval J., Gollub M., Wu A. (2023). Sustained organ preservation in patients with rectal cancer treated with total neoadjuvant therapy: Long-term results of the OPRA trial. J. Clin. Oncol..

[21] Yu S., Mamtani R., O’Hara M.H., O’Dwyer P.J., Margalit O., Giantonio B.J., Shmueli E., Reiss K.A., Boursi B. (2021). Comparative Effectiveness of Total Neoadjuvant Therapy Versus Standard Adjuvant Chemotherapy for Locally Advanced Rectal Cancer. Clin. Color. Cancer.

[22] Turfa R., Alawabdeh T., Naser A., Alamro Y., Albliwi M., Almasri S., Al Qazakzeh A., Shattal M.A., Dabous A., Amarin R. (2023). Comparing real-world outcomes of total neoadjuvant treatment and CRT at a tertiary medical center. Front. Oncol..

[23] Goffredo P., Quezada-Diaz F.F., Garcia-Aguilar J., Smith J.J. (2022). Non-Operative Management of Patients with Rectal Cancer: Lessons Learnt from the OPRA Trial. Cancers.

[24] Velayati A., Kalmuk J., Roubal K., Wolf B., Misniakiewicz J., Donahue C., George V., Lupak O. (2023). Clinical outcomes of patients who underwent total neoadjuvant therapy (TNT) for locally advanced rectal cancer (LARC): A single center experience. J. Clin. Oncol..

[25] Garcia-Aguilar J., Patil S., Gollub M.J., Kim J.K., Yuval J.B., Thompson H.M., Verheij F.S., Omer D.M., Lee M., Dunne R.F. (2022). Organ Preservation in Patients With Rectal Adenocarcinoma Treated With Total Neoadjuvant Therapy. J. Clin. Oncol..

[26] Cercek A., Roxburgh C.S.D., Strombom P., Smith J.J., Temple L.K.F., Nash G.M., Guillem J.G., Paty P.B., Yaeger R., Stadler Z.K. (2018). Adoption of Total Neoadjuvant Therapy for Locally Advanced Rectal Cancer. JAMA Oncol..

[27] Cercek A., Lumish M., Sinopoli J., Weiss J., Shia J., Lamendola-Essel M., El Dika I.H., Segal N., Shcherba M., Sugarman R. (2022). PD-1 Blockade in Mismatch Repair-Deficient, Locally Advanced Rectal Cancer. N. Engl. J. Med..

[28] Shamseddine A., Zeidan Y.H., Kreidieh M., Khalifeh I., Turfa R., Kattan J., Mukherji D., Temraz S., Alqasem K., Amarin R. (2020). Short-course radiation followed by mFOLFOX-6 plus avelumab for locally-advanced rectal adenocarcinoma. BMC Cancer.

[29] Papke D.J., Yurgelun M.B., Noffsinger A.E., Turner K.O., Genta R.M., Redston M. (2022). Prevalence of Mismatch-Repair Deficiency in Rectal Adenocarcinomas. N. Engl. J. Med..

